# TBC-YOLOv7: a refined YOLOv7-based algorithm for tea bud grading detection

**DOI:** 10.3389/fpls.2023.1223410

**Published:** 2023-08-17

**Authors:** Siyang Wang, Dasheng Wu, Xinyu Zheng

**Affiliations:** ^1^ College of Mathematics and Computer Science, Zhejiang A&F University, Hangzhou, China; ^2^ Key Laboratory of State Forestry and Grassland Administration on Forestry Sensing Technology and Intelligent Equipment, Hangzhou, China; ^3^ Key Laboratory of Forestry Intelligent Monitoring and Information Technology of Zhejiang, Hangzhou, China

**Keywords:** YOLOv7, contextual transformer, BiFPN, CA, SIoU, tea bud grading detection

## Abstract

**Introduction:**

Accurate grading identification of tea buds is a prerequisite for automated tea-picking based on machine vision system. However, current target detection algorithms face challenges in detecting tea bud grades in complex backgrounds. In this paper, an improved YOLOv7 tea bud grading detection algorithm TBC-YOLOv7 is proposed.

**Methods:**

The TBC-YOLOv7 algorithm incorporates the transformer architecture design in the natural language processing field, integrating the transformer module based on the contextual information in the feature map into the YOLOv7 algorithm, thereby facilitating self-attention learning and enhancing the connection of global feature information. To fuse feature information at different scales, the TBC-YOLOv7 algorithm employs a bidirectional feature pyramid network. In addition, coordinate attention is embedded into the critical positions of the network to suppress useless background details while paying more attention to the prominent features of tea buds. The SIOU loss function is applied as the bounding box loss function to improve the convergence speed of the network.

**Result:**

The results of the experiments indicate that the TBC-YOLOv7 is effective in all grades of samples in the test set. Specifically, the model achieves a precision of 88.2% and 86.9%, with corresponding recall of 81% and 75.9%. The mean average precision of the model reaches 87.5%, 3.4% higher than the original YOLOv7, with average precision values of up to 90% for one bud with one leaf. Furthermore, the F1 score reaches 0.83. The model’s performance outperforms the YOLOv7 model in terms of the number of parameters. Finally, the results of the model detection exhibit a high degree of correlation with the actual manual annotation results ( $R^{2}$ =0.89), with the root mean square error of 1.54.

**Discussion:**

The TBC-YOLOv7 model proposed in this paper exhibits superior performance in vision recognition, indicating that the improved YOLOv7 model fused with transformer-style module can achieve higher grading accuracy on densely growing tea buds, thereby enables the grade detection of tea buds in practical scenarios, providing solution and technical support for automated collection of tea buds and the judging of grades.

## 1 Introduction

In 2022, China's tea plantations covered an area of 49,954,000 square meters, with a 2.03% increase compared to the previous year, and tea production reached a total of 3.810 million tons (Mei and Zhang, 2022). The total global tea production has maintained growing trend. With the growing tea cultivation scale and increasing tea varieties, consumers have put forward higher requirements for tea harvesting as well as processing techniques. Meanwhile, it is noteworthy to efficiently detect tea product quality should be further improved to meet the demand for larger-scale premium tea products. The grade of tea buds is an essential factor in determining the economic value of the product. During the processing of tea sprouts picking, different picking points can lead to different tea quality (Yang et al., 2021). Typically, the number of leaves is one of the important factors in determining the quality level of tea, such as having one bud with one leaf, and one bud with two leaves. Precise grading of tea buds is essential before implementing selective picking strategies (Xu et al., 2022). Tea bud target detection in complex backgrounds poses challenges due to small size of tea buds, similarity between tea buds and tea leaves, and blending between tea buds and their environment. Moreover, occlusion of tea leaves and tea buds complicate the detection. Variations in lighting and environment increase noise and interference, which also adds additional challenges. Despite previous efforts, persistent challenges require further advancements for effective detection. Yang et al. (2019) have proposed a residual network block structure in the down-sampling section of the YOLOv3 algorithm and replaced the fully connected layer with a 1×1 convolution operation, resulting in an accuracy rate exceeding 90% for identifying high-quality tea buds with various poses and types in the case where the image background is relatively simple and the dataset contains only one tea bud target. In a similar vein, Li S, et al. (2022) used the YOLOv3 algorithm and spatial pyramid module to compress the model scale by channel pruning and hierarchical pruning algorithms, significantly improving detection speed in the practical environment. However, the task only distinguished the angle of tea buds. Therefore, improving the accuracy of detection and grading of tea buds based on complex background becomes the main purpose of this paper. The contributions of this study are as follows:

(1) A tea bud detection model called TBC-YOLOv7 is proposed to achieve accurate identification and quality grading of tea buds.
(2) A transformer-based architectural design was employed in the YOLOv7 network to enhance visual representations using the rich contextual information of adjacent keys to facilitate self-attentive learning mechanisms.
(3) The bidirectional feature pyramid network (BiFPN) is used to fuse features in both directions, integrating local and global tea buds’ features.
(4) The network combines the coordinate attention (CA) module to reinforce the ability to extract feature information.
(5) The SIOU loss function is preferred as the bounding box loss function of the network to obtain higher detection performance and prediction results.

The rest of this study is organized as follows: section 2 presents an overview of relevant methods in the field of tea bud identification. Next, section 3 describes the experimental methods and principles in detail, mainly including the principles of YOLOv7 and the proposed method of TBC-YOLOv7. Subsequently, section 4 presents and analyzes the experimental results and shows the comparison and discussion of the proposed method with related popular methods. In addition, section 5 discusses the difficulties encountered in the related work in this study as well as the limitations. Finally, section 6 concludes the work of this study and provides an outlook for future research work.

## 2 Related work

Numerous scholars have analyzed and compared various methods for image processing technology in tea bud identification and positioning (Motokura et al., 2020), tea pest and disease detection (Han et al., 2019), tea variety identification and quality detection (Yan et al., 2022). For the problem of tea bud recognition and detection, scholars around the world have proposed corresponding various methods, mainly including traditional machine vision and deep learning methods that have emerged in recent years. The conventional machine vision methods extract the feature information of the target based on the differences in posture, color, and texture features among tea buds, tender leaves, and old leaves, to achieve the discrimination of tea targets. Zhang et al. (2021) combined the blue component with the green component to obtain a new combined component grey-scale image by adaptive processing. The segmentation of tea bud targets was achieved by a segmentation linear transform and an improved watershed algorithm. However, the above-mentioned traditional machine vision methods still cannot be applied in complex natural environment scenarios, making it difficult to meet more practical requirements (Kamilaris and Prenafeta-Boldú, 2018). With the widespread use of deep convolutional neural networks (Picon et al., 2019), R-CNN (Girshick et al., 2013), Fast R-CNN (Girshick, 2015), Faster R-CNN (Ren et al., 2015), SSD (Liu et al., 2015), and YOLO series algorithms (Chen et al., 2022; Wu et al., 2022; Subedi et al., 2023) have been proposed for target detection tasks. Due to the fact that deep learning algorithms can not only accurately identify target categories but also quickly label their locations, they can more effectively solve the recognition tasks related to tea leaves in complex environments (Barbedo, 2018). Li Y. et al. (2023) combined the improved YOLOv5 algorithm with the Hungarian matching algorithm and the Kalman filter algorithm for tracking and monitoring of tea bud targets as well as yield estimation. A multi-objective continuous sorting model for machine-picked tea leaves was developed by (Zhang et al., 2023). Xue et al. (2023) solved the tea pest task by improving the YOLOv5 network and introducing the Global Context Network. While the YOLOv7 algorithm (Wang et al., 2022) proposed in 2022 has outperformed various target detectors such as YOLOX (Ge et al., 2021), YOLOv4 (Bochkovskiy et al., 2020), YOLOv5 (Qiao et al., 2023) in terms of speed and accuracy, however, research on the application for detecting tea buds is still limited (Jiang et al., 2022).

Currently, Transformer-based target detection algorithms have garnered significant attention in the realm of deep learning frameworks. Transformer can exhibit formidable modeling capabilities and parallel computing prowess. Li et al. (2023) conducted a comprehensive review of Transformer-based target detection algorithms, categorizing them into four aspects: feature learning, target estimation, label matching strategy and algorithm application. A comparative analysis was performed between Transformer-based algorithms and convolutional neural network (CNN) algorithms in target detection tasks. Compared to CNNs, Transformers possess a larger perceptual field, a more flexible weight-setting mechanism, and the ability to model global features. The global interaction capability of Transformers can be combined with the localized features of CNNs to enrich feature diversity. In 2020, Carion et al (2020) introduced a novel Transformer-based target detection framework called DETR (Detection Transformer). DETR employed a set prediction approach, enabling end-to-end training without reliance on prior design choices. However, challenges such as slow convergence and subpar results in small target detection were observed. Consequently, a multitude of improved algorithms based on DETR have emerged (Li M. et al., 2023). The remarkable performance of Transformer-based target detection algorithms on standard datasets has prompted researchers to explore their application in various real-world scenarios, offering novel solutions across diverse domains (Li Y, et al., 2022). This fusion has demonstrated applicability to a wide range of vision tasks and exhibits great potential.

## 3 Materials and methods

### 3.1 YOLOv7 baseline network structure

**Figure 1:**
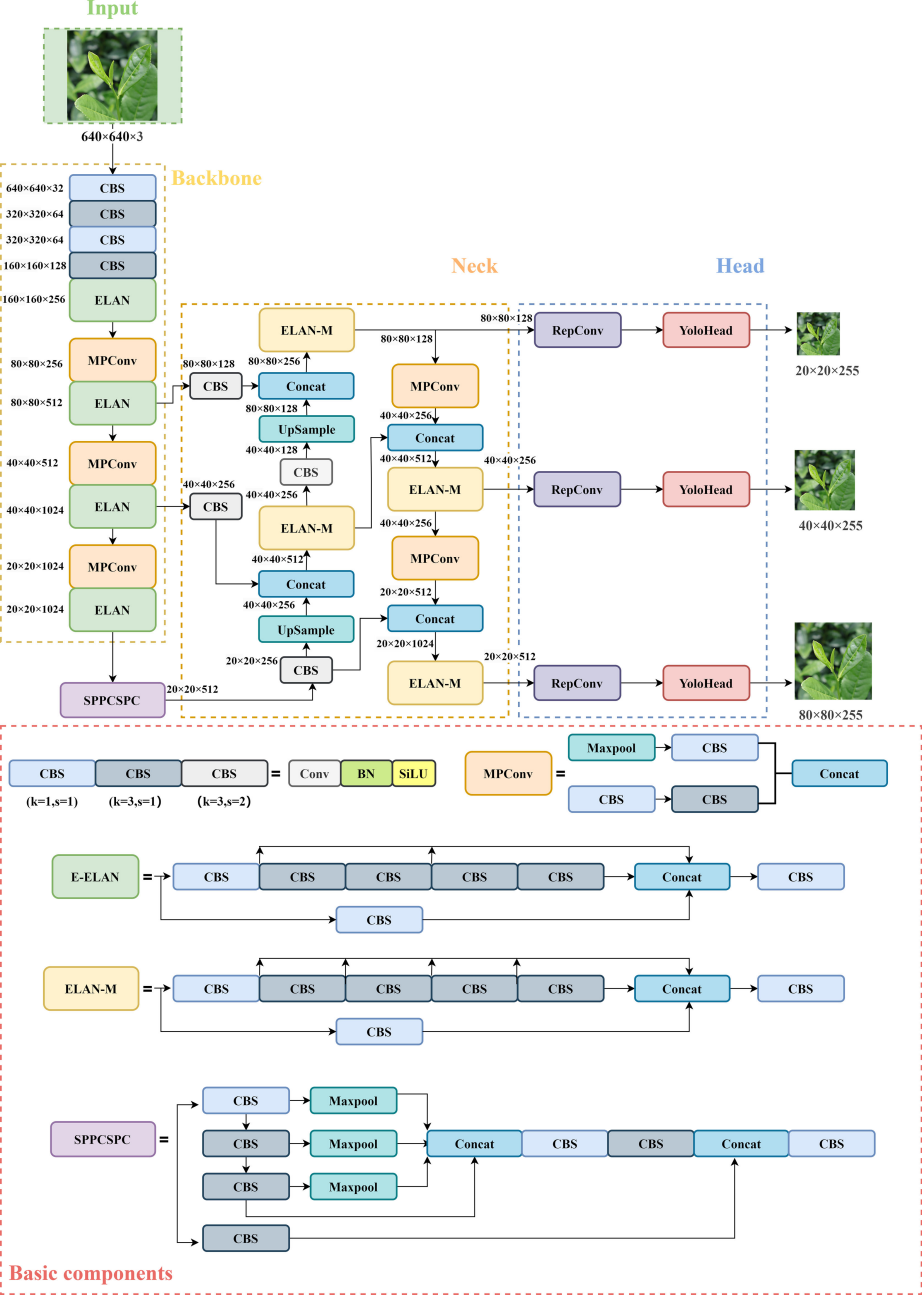
YOLOv7 network structure diagram. The basic CBS module has three colors, representing different convolutional kernels size and strides represented by k and s.

In the algorithms of YOLO series, Wang et al. (2022) proposed the YOLOv7 algorithm in 2022 with the structure of 4 parts (as shown in **Figure 1**): Input, Backbone, Neck, and Head prediction. The improving strategies of YOLOv7 is as follows: (1) The extended efficient layer aggregation network (ELAN) serves as its network architecture, using group convolution to increase the cardinality of the added features and enhance the features learned by various feature maps. (2) Model scaling for concatenation-based models, which generates different model sizes by adjusting the properties of the model. (3) The network architecture is augmented with model re-parameterization convolution to provide more gradient diversity (Ding et al., 2021). (4) Dynamic label allocation strategy integrating cross-grid search of YOLOV5 and matching strategy of YOLOX. The most suitable prior bounding box is adaptively and precisely selected by increasing the number of positive samples. (5) An auxiliary head training method is used to improve accuracy by increasing training costs without affecting the inference time.

The backbone feature extraction network of YOLOv7 consists of several CBS modules, ELAN structures, and MP convolutional layers (Praveen et al., 2022). The CBS module consists of a convolutional layer, a batch normalization layer, and a SiLU activation function. The ELAN structure continuously enhances the network’s learning ability by controlling the shortest and longest gradient path, thereby effectively extracting features. The down-sampling structure is designed as an MP convolutional layer consisting of a maximum pooling layer and a convolutional layer for parallel feature extraction and compression. The SPPCSP structure is utilized in the last layer of the backbone network to introduce a sizeable residual branch to help optimize feature extraction, reduce computation and expand the receptive field. The enhanced feature extraction network in the Neck still follows PANet structure in the YOLOv5 network, which performs up-sampling and down-sampling operations on the features at different scales obtained from the backbone to achieve feature fusion (Liu et al., 2018). The Head adjusts the number of image channels for three different scales of features output from PANet using the re-parameterization convolution structure, and then passes through 1 × 1 convolution to predict confidence, categories, and anchor boxes.

### 3.2 Improved YOLOv7 structural design (TBC-YOLOv7)

**Figure 2:**
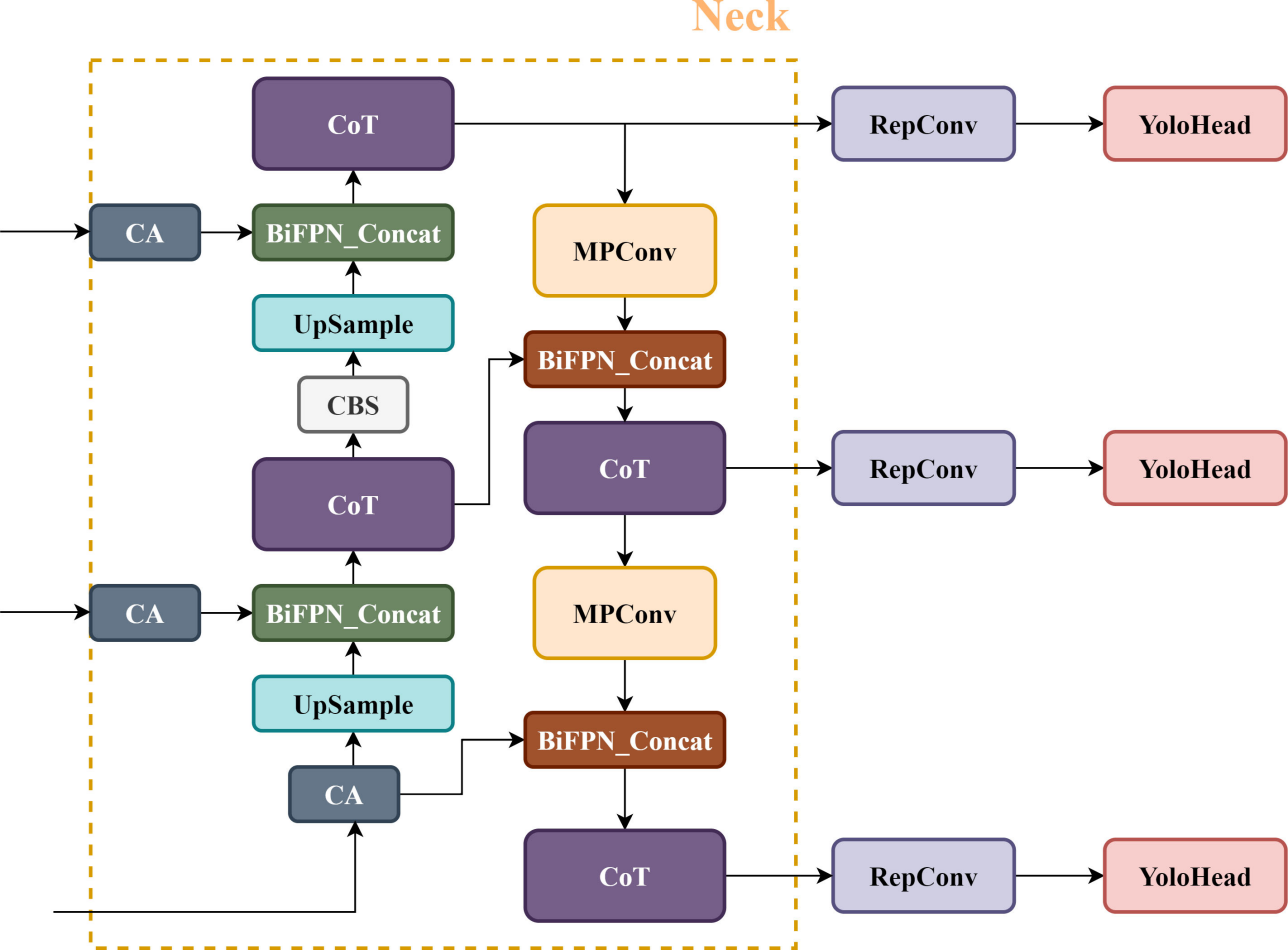
Neck components of the TBC-YOLOv7 network.

To improve the tea grading and picking efficiency, we propose the TBC-YOLOv7 model (shown as in **Figure 2**) in this paper. The details of improvements are described as follows: (1) Our approach employs a transformer-based architecture design in network, which integrated the contextual transformer (CoT) module into the YOLOv7 network to replace the ELAN-M structure of the Neck part. Specifically, the CoT module fully utilizes the contextual information between the input keys to guide the learning of the dynamic attention matrix. By simultaneously capturing two kinds of spatial contexts, it efficiently promotes self-attention learning, strengthens the representative ability of the output feature map, and optimizes the relationship between the global information of the network. (2) To efficiently fuse multi-scale features, the BiFPN structure replaces the original approach of fusing features in YOLOv7. This structure optimizes channel relationships and long-term dependencies by using precise location information for encoding, producing better-perceived location information than the original structure. (3) To address the issue of direction mismatch between the real box and prediction box, the original CIOU (complete IOU) loss function is improved to the SIOU loss function which focuses on the change of the angle vector between the ground truth box and the prediction box, and fusing direction information to improve the convergence speed of the network. (4) To replace the three convolutional blocks on the output feature layer of the backbone network with the CA mechanism module, it can utilize more shallow features to enrich the expressiveness of the feature map. By avoiding extracting redundant features and reducing the weight of non-significant features, making the model more accurate and sensitive in identifying the positions of interested targets.

### 3.3 Contextual transformer networks

Transformer has shown remarkable ability for capturing long-distance features in natural language processing. We attempt to migrate the transformer module to computer vision to more efficiently handle long-distance inputs and enable training parallelization through self-attention learning of feature vectors at different spatial locations in images (Yin et al., 2020). By conducting self-attention learning in transformer, the dependence on external information is reduced, resulting in a greater focus on the internal correlation of data or features (Vaswani et al., 2017). The attention matrix is calculated using the independent query-key pairs in the self-attention module, which combines the strong modeling capability of the transformer module and the critical visual feature signals to achieve better detection results (Carion et al., 2020).

**Figure 3:**
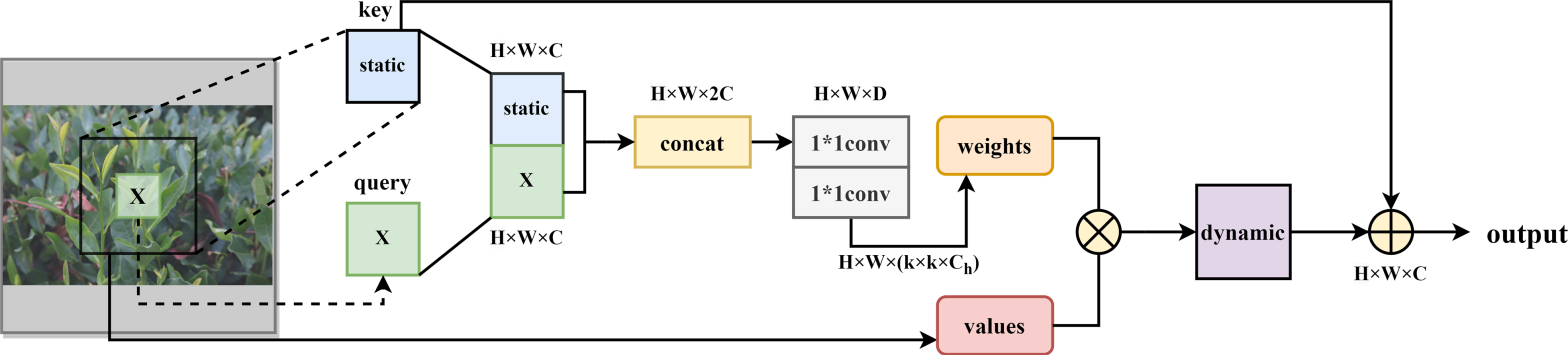
CoT module structure diagram.


However, the traditional self-attention mechanism only considers the local relationship matrix by using location information, while ignoring the contextual information among the nearest neighbors, which severely limits the expressiveness of two-dimensional feature maps (Linsley et al., 2018). Therefore, the CoT module (as shown in **Figure 3**) makes full use of the contextual information between neighbor keys to capture global information to obtain a larger receptive field, which is superior to the classical local self-attention (Li et al., 2021). In the CoT module, as shown in equations (1) and (2), all neighbor keys in the 2-D feature input graph are first contextually encoded by group convolution to generate the key feature $K^{1}$ of the context among local neighbors, after that $K^{1}$ is used as the static contextual representation of the input X. Furthermore, conditioned on the concatenation of the $K^{1}$ and the queries Q, and then the dynamic multi-head attention matrix A is obtained by two consecutive 1×1 convolutions ( $W_{\theta }$ with ReLU activation function and $W_{\delta }$ without activation function), which is multiplied by the input value V for further aggregation. In equation (2), the ⊛ denotes local matrix multiplication, in which a weighted feature map $K^{2}\;$ is obtained to represent the dynamic interaction features between the inputs. 

(1) $$\begin{array}{c} A=\left[K^{1},Q\right]W_{\theta }W_{\delta } \end{array}$$

(2) $$K^{2}=V⊛A$$

Finally, the static and dynamic contextual representations $K^{1}$ and $K^{2}$ are linearly fused as the final output. This module substitutes the convolutional blocks of the same size in the network backbone of the vision scenario to produce a network backbone with transformer characteristics style. The CoT module is capable of integrating contextual information mining between neighbor keys and self-attentive learning of 2-D feature maps into a unified architecture, avoiding introducing additional branching structures for context mining with a reasonable parameter budget and enhanced visual representation capabilities.

### 3.4 The bidirectional feature pyramid network

**Figure 4:**
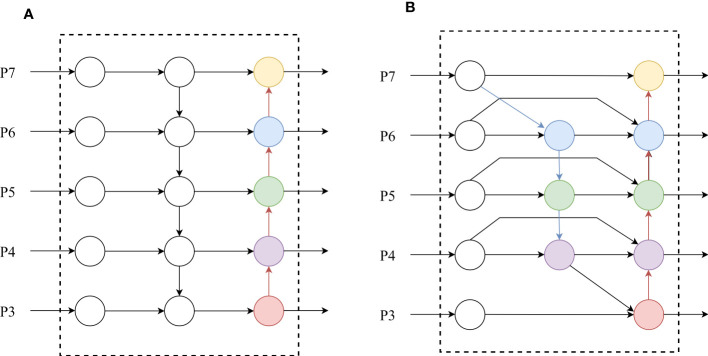
PANet structure diagram **(A)** and BiFPN structure diagram **(B)**.

Currently, the PANet used in YOLOv7 is a one-way fusion feature approach. However, the environment for tea bud detection often makes it difficult to extract significant features, and the PANet structure (as shown in **Figure 4A** lacks original feature information in the extracted information, which easily lead to deviations in training and learning. Therefore, we use a cascading method to integrate the BiFPN structure (as shown in **Figure 4B**) into the enhanced feature extraction network of YOLOv7. The BiFPN increases cross-scaled connections based on the PANet structure and simplifies nodes with only one input edge and one output edge. Specifically, the BiFPN designs a top-down path from p7 to p3 to transfer semantic information of high-level features to the lower levels and a bottom-up path from p3 to p7 to transfer location information of bottom-level features to the higher levels. Additionally, the BiFPN adds a connection from p4 to p6 that directly connects input and output nodes of the same layer striding over intermediate layers to achieve deeper fusion (Zhong et al., 2021).

Resolutions from different feature layers may result in different contributions to output. The high-resolution shallow feature maps have richer detail information, but less semantic feature information. The network retains more shallow semantic information in the tea bud images without losing too much deep location information while integrating local and global features of tea buds, thus achieving accurate detection of tea buds of different sizes and occlusion.

### 3.5 Loss function

The loss function consists of three components: localization loss, confidence loss, and classification loss. Since tea bud detection is a dense detection task, the loss function is needed to consider the aggregation of metrics of bounding box regression, such as the distance, overlap area, and aspect ratio of the prediction and ground truth boxes when the model locates the target. CIOU loss function used by the YOLOv7 network relies heavily on aggregating bounding box regression metrics without considering the desired mismatch direction between the ground truth boxes and prediction boxes (Zheng et al., 2020). SIOU loss function redefines the penalty metric so that the prediction box to move to the nearest axis quite quickly, effectively reducing the total number of degrees of freedom. It also considers the vector angle between the desired regressions to make the prediction bounding box more stable during the training process, this consideration can greatly speed up the training convergence process (Gevorgyan, 2022).

**Figure 5:**
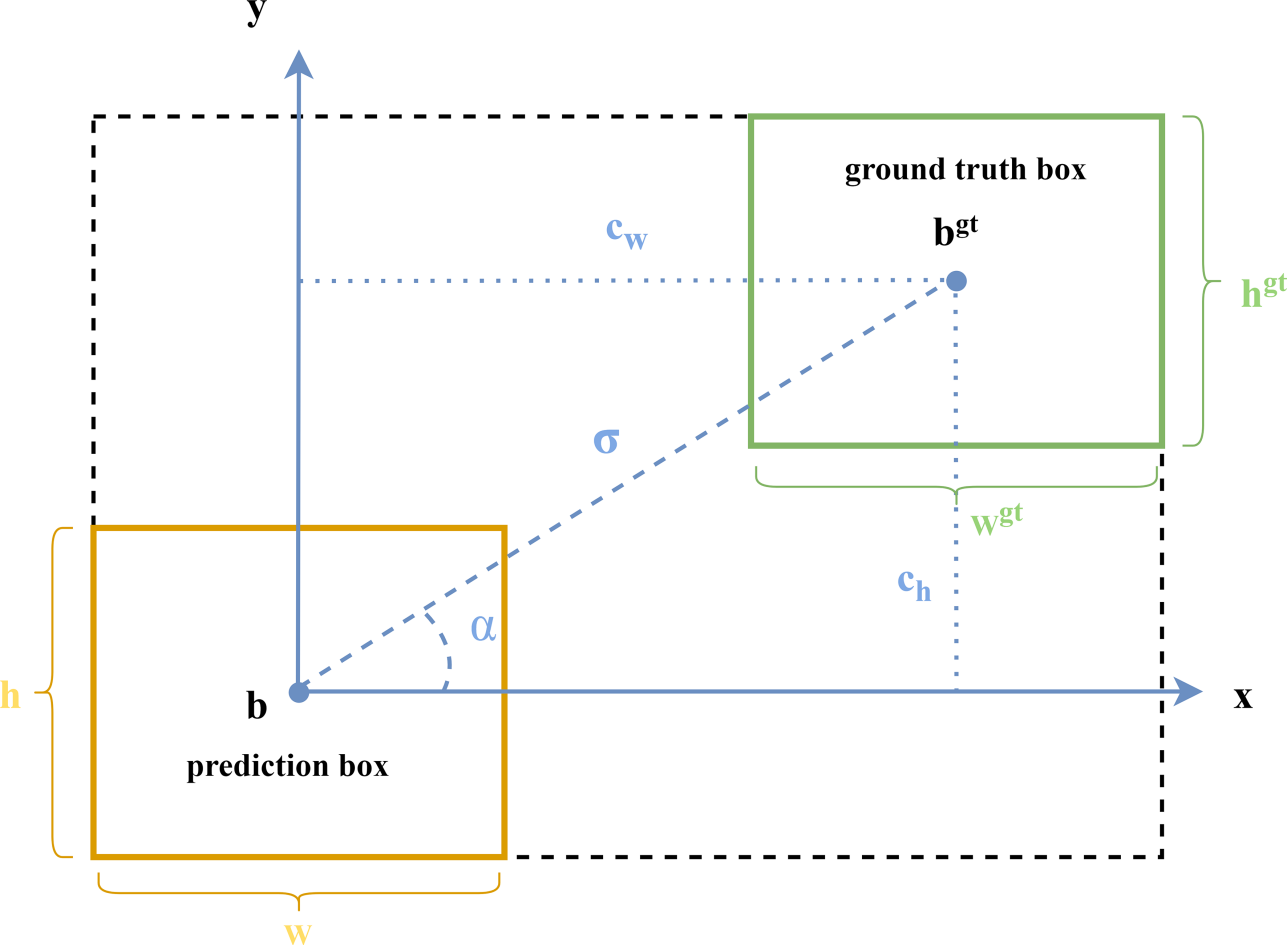
SIOU: schematic diagram of loss function calculation cost.

The SIOU contains four components: Angle cost, Distance cost, Shape cost, and IOU cost. The SIOU loss function is illustrated (as shown in **Figure 5**) as follows:

The component of Angle cost [as shown in equation (3)] enables the prediction bounding box to preferentially reach the x or y axis depending on the minimum distance from the x axis or y axis, and then continue to approach the ground truth box along the preferable axis, which can speed up the distance calculation between the two boxes. 


(3) $$\begin{array}{c} \Lambda =1-2*{\text{sin}}^{2}\left(\text{arcsin}\left(\frac{c_{h}}{\sigma }\right)-\frac{\pi }{4}\right) \end{array}$$

Where, 


(4) $$\begin{array}{c} \sigma =\sqrt{{\left(b_{c_{x}}^{gt}-b_{c_{x}}\right)}^{2}+{\left(b_{c_{y}}^{gt}-b_{c_{y}}\right)}^{2}} \end{array}$$

(5) $$\begin{array}{c} c_{h}=\max\left(b_{c_{y}}^{gt},b_{c_{y}}\right)-\min\left(b_{c_{y}}^{gt},b_{c_{y}}\right) \end{array}$$

$\left(b_{c_{x}},b_{c_{y}}\right)$ , $\left(b_{c_{x}}^{gt},b_{c_{y}}^{gt}\right)$ indicate the coordinate positions of the prediction box and the ground truth box, respectively.

In addition, the distance cost describes the distance between the two centroids of prediction box and the ground truth box. The SIOU adjust the minimum outer rectangle of the ground truth box and the prediction box according to the Angle cost when calculating the Distance cost [as shown in equation (6)]. 


(6) $$\begin{array}{c} \Delta =\Sigma_{t=x,y}\left(1-e^{-\rho t\gamma }\right) \end{array}$$

Where,


(7) $$\begin{array}{c} \rho_{x}={\left(\frac{b_{c_{x}}^{gt}-b_{c_{x}}}{c_{w}}\right)}^{2} \end{array}$$

(8) $$\rho_{y}={\left(\frac{b_{c_{y}}^{gt}-b_{c_{y}}}{c_{h}}\right)}^{2}$$

(9) γ=2−Λ

$c_{h}$ , $c_{w}$ represent the width and height of the minimum outer rectangle between the center of the ground truth box and the prediction box, respectively.

The shape cost is defined as the equation (10). The shape cost considers the aspect ratio between the target box and the prediction box to make their shapes more similar. Where, θ indicates the extent of concern for controlling shape loss. By selecting the suggested aspect ratio between the ground truth box and the prediction box to avoid reducing the movement of the prediction box due to excessive focus on shape loss in the calculation. 


(10) $$\begin{array}{c} \Omega =\sum_{t=w,h}{\left(1-e^{-\omega_{t}}\right)}^{\theta } \end{array}$$

Where,


(11) $$\begin{array}{c} \omega_{w}=\frac{\left|w-w^{gt}\right|}{\max\left(w,w^{gt}\right)} \end{array}$$

(12) $$\begin{array}{c} \omega_{h}=\frac{\left|h-h^{gt}\right|}{\max(h,h^{gt})} \end{array}$$

w,h indicate the width and height of the prediction box, $w^{gt},h^{gt}$ indicate the width and height of the ground truth box.

The IOU cost is calculated by equation (13). 


(13) $$\begin{array}{c} \text{IOU}\left(\text{A},\text{ B}\right)=\frac{\text{A}\cap^{\;}\text{B}}{\text{A}\cup^{\;}\text{B}} \end{array}$$

Where, A is the prediction box, B is the ground truth box, and IOU indicates the overlap rate of the prediction box with the ground truth box.

Therefore, the SIOU loss function is calculated by equation (14): 


(14) $$\begin{array}{c} Los{\text{s}}_{SIOU}=1-\text{IOU}+\frac{\text{Δ}+\text{Ω}}{2} \end{array}$$

### 3.6 Coordinate attention

The main purpose of the attention mechanism is to enable the model to acquire adaptive attention to focus on the more critical parts of the image. The usual attention mechanism is implemented by converting feature tensors into individual feature vectors through global pooling, but it is prone to ignore spatial location information. CA not only can capture inter-channel information but also capture information about direction-perception and position-perception, which achieves effective separation of the target area and background in the image (Hou et al., 2021).

**Figure 6:**
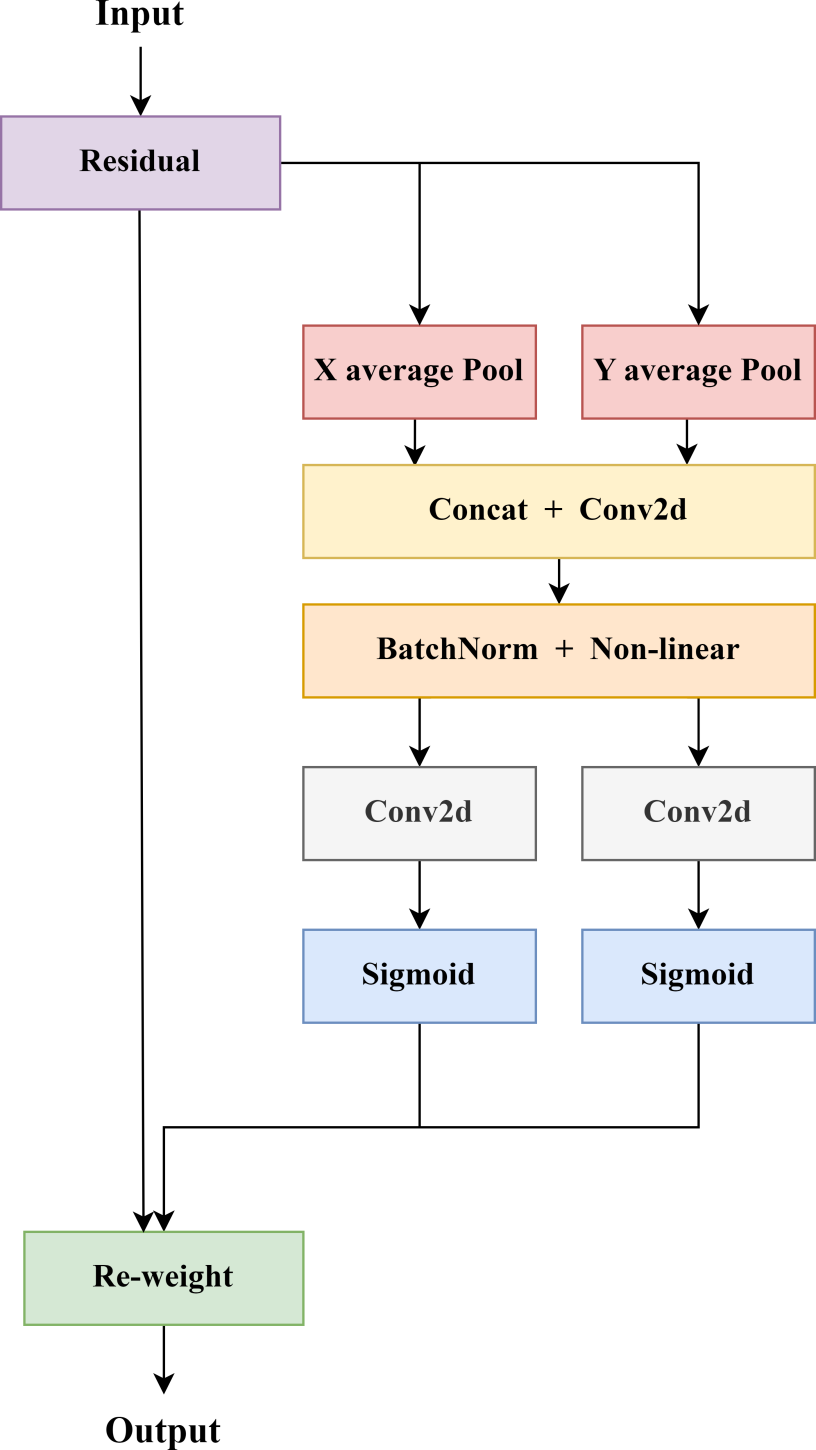
Coordinate attention mechanism structure diagram.

CA module utilizes accurate location information for learning the relationship between channels, performing global average pooling in the x and y directions, resulting in two one-dimensional feature encoding vectors in the horizontal and vertical directions. Afterwards a pair of orientation-aware feature maps were generated by embedding of coordinate information to obtain accurate location information (as shown in **Figure 6**). The two generated feature maps are cascaded in the spatial dimension, and a 1×1 convolutional transformation function is used to reduce the number of channels of the feature vectors, thereby reducing the complexity of the model. After batch normalization and nonlinear activation function processing, the intermediate feature map for spatial information encoding is obtained. Meanwhile, the intermediate feature map is decomposed into independent feature tensors in horizontal and vertical directions along the spatial dimension. Furthermore, the number of feature channels is adjusted to the same number as the initial input features by the 1×1 convolutional transformation and nonlinear activation functions, respectively. Finally, the spatial information of the two directions is fused in a weighted manner, and the sigmoid activation function is applied to obtain the attention weights in two different directions, and then multiplied by the input feature map for the final output.

The feature maps obtained by the CA module can capture long-range dependencies along one spatial direction while preserving accurate spatial information along another direction. Consequently, the model exhibits improved robustness even in the complex environment. By complementarily applying the feature maps at different levels, the representation of tea buds' important feature location information is enhanced, enabling the network to better focus on crucial locations from a larger area.

## 4 Experiments and results analysis

### 4.1 Data acquisition and pre-processing

**Figure 7:**
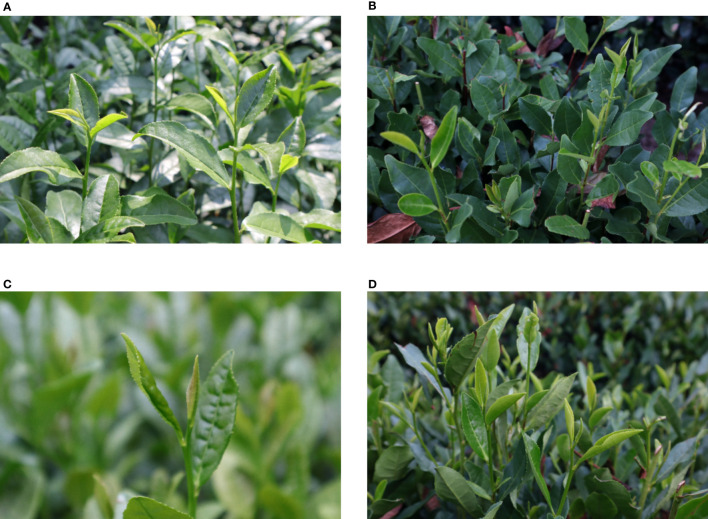
Tea bud samples under different environmental conditions: intense light environment **(A)**; weak light environment **(B)**; with a single-target sample **(C)**; with multiple intensive target samples **(D)**.

Experimental data were collected at the China Tea Museum campus in west lake District, Hangzhou, Zhejiang Province. A single-micro camera Canon M50 was utilized for image acquisition of tea buds. To increase the richness of the background in the images and improve the model’s generalization ability, various poses of tea buds were captured under diverse natural environmental conditions. The natural growth environment is simulated by photographing the different conditions of the germination environment. We carried out image acquisition in mid-July and mid-September 2022. The experimental data included summer and autumn teas, choosing two time periods with different light intensities: 7:00-9:00 and 13:00-15:00, with a shooting angle range of 30° and random shooting distance, and an image resolution of 6000*4000 pixels. Ultimately, a total of 557 original images were eventually collected, and four samples of the dataset are shown in **Figure 7**.

**Figure 8:**
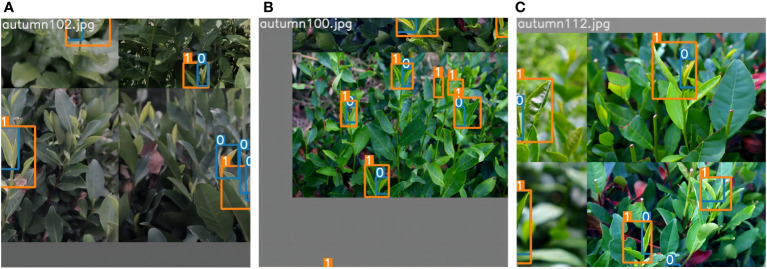
Tea image’s data enhancement effect: **(A–C)** represent the data enhancement effect after different methods. Use the numbers 0 and 1 to label the different grades of tea buds.

**Figure 9:**
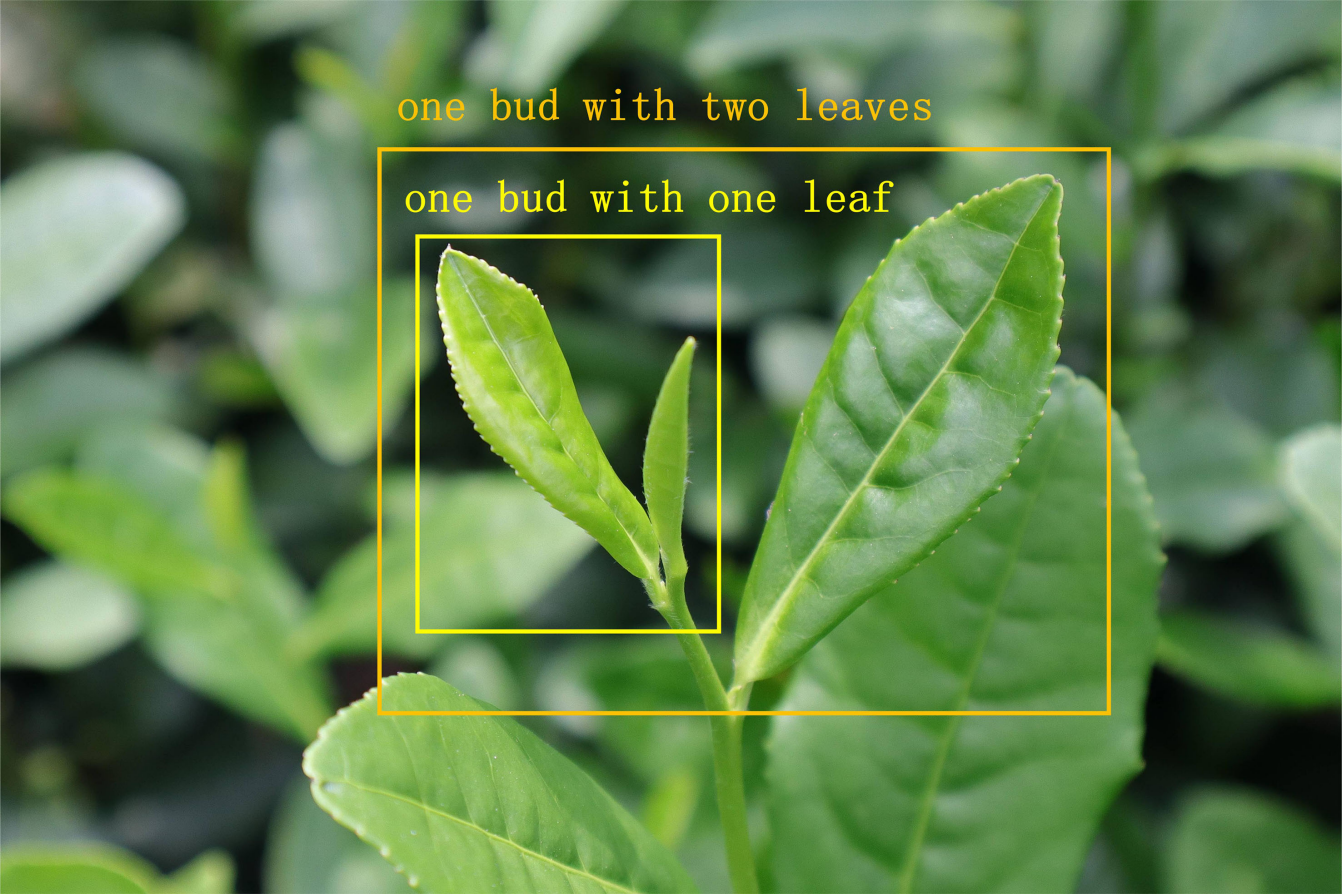
Tea bud classification criteria. One bud with one leaf is denoted by “BOL” and one bud with two leaves is denoted by “BTL”.

Different background environments can cause disturbing factors in recognizing tea bud features. To address this issue, online data augmentation methods such as random image scaling and mosaic data augmentation were employed to the research during the training process (as shown in **Figure 8**). The mosaic data augmentation method first stitches the four images to be detected into one image by random scaling as training data. Subsequently, the entire image is input into the neural network for detection. Additionally, the hue is randomly adjusted by 1.5%, the saturation by 70% and the value by 40% of the image. Moreover, the image is set to flip up and down with a 50% probability, and the degree of random is set to 20%. Meanwhile, the image is randomly scaled to 90%, and 15% of the images are randomly selected for copying and pasting. Therefore, this data augmentation method could enrich the background of the detection target, and effectively enhance the model’s detection accuracy under interference conditions. The tea buds are graded into two categories, in which one bud with one leaf is denoted by “BOL” and one bud with two leaves is denoted by “BTL”, as shown in **Figure 9**. The annotation tool is utilized to manually annotate the dataset and mark the location of the two categories of tea buds with rectangular boxes. Subsequently, the dataset is randomly divided into the train set and the test set by the ratio of 9:1. Specifically, the number of images, the number of BOL, and the number of BTL are shown in **Table 1**.

**Table 1:** Tea bud data set.

Dataset	Number of images	Number of BOL	Number of BTL	Total number of targets
Train set	502	1635	1324	2959
Test set	55	194	166	360
Total	557	1829	1490	3319

### 4.2 Model experimental environment and methods

The configuration of environment parameters for the experimental models is shown in **Table 2**, which can help to transfer knowledge of common features using convolutional layers and support fine-tuning strategies to enhance the learning stability and generalization ability of the network.

**Table 2:** Experimental environment.

Configuration	Parameter
CPU	Inter(R) Core (TM) i5-12490F
GPU	NVIDIA GTX3060
OS	Windows10
Framework	PyTorch
CUDA	cuda11.6

The hyperparameter settings of the models are as follows: the input image size of the network is 640×640×3, the optimizer is Adam, the initial learning rate is set to 0.01, and the learning rate is dynamically adjusted by utilizing the cosine function (Srinivasu et al., 2022). During training, the learning rate is saturated near the 290th epoch with a learning rate of approximately 0.001. In addition, the momentum factor is set to 0.937, the weight decay is set to 0.0005, the number of epochs is 300. Moreover, the training phase is divided into frozen and unfrozen phases, in which the feature extraction network does not change during the frozen phase, thereby reducing the occupation of memory and improving training efficiency.

### 4.3 Model evaluation metrics

This study employs a series of performance indicators to evaluate the efficacy of the proposed approach. These indicators include precision, recall, and mean average precision (mAP). Precision refers to the ratio of correctly identified positive samples to the total number of predicted positive samples. Recall corresponds to the ratio of correctly identified true positive samples to the overall number of measured positive samples. Where True Positive (TP) denotes the number of correctly predicted buds, False Positive (FP) denotes the number of incorrectly predicted buds, and False Negative (FN) represents the number of undetected buds. The average precision (AP) is the average of the precision values over the area under the Precision-Recall curve and the coordinate axes. The mAP is the average of the AP values for each detected category. In addition, frames per second (FPS), and floating point of operations (FLOPs) are used to evaluate the size of the model, the speed of detection of the model and the computational cost. The definitions of formulas are as follows: 


(15) $$\begin{array}{c} precision=\frac{TP}{TP+FP} \end{array}$$

(16) $$\begin{array}{c} Recall=\frac{TP}{TP+FN} \end{array}$$

(17) $$\begin{array}{c} F1=2\frac{precision\times Recall}{precision+Recall} \end{array}$$

(18) $$\begin{array}{c} AP=\int_{0}^{1}P\left(r\right)dr \end{array}$$

(19) $$\begin{array}{c} mAP=\frac{\sum_{i=1}^{S}AP_{i}}{S} \end{array}$$

In Equation (19), S is the number of detected categories, $AP_{i}$ represents the accuracy rate of the i th category. Moreover, root mean squared error (RMSE) and coefficient of determination $\left(R^{2}\right)$ are also introduced to evaluate the metrics of regression effect between predicted values and measured values. RMSE is the mean value of the square root of the error between the predicted and actual values; a higher RMSE indicates superior model performance; $R^{2}$ is the square of the correlation coefficient between the actual result and the predicted values constructed by the model, indicating the degree of similarity between the predicted and actual values. The definitions are as follows: 


20 $$\begin{array}{c} R^{2}=1-\frac{\sum_{i}{\left({\hat{y}}_{i}-y_{i}\right)}^{2}}{\sum_{i}{\left({\bar{y}}_{i}-y_{i}\right)}^{2}} \end{array}$$

(21) $$\begin{array}{c} RMSE=\sqrt{\frac{1}{n}\sum_{i=1}^{m}w_{i}{\left(y_{i}-{\hat{y}}_{i}\right)}^{2}} \end{array}$$

### 4.4 Analysis of experimental results

**Figure 10:**
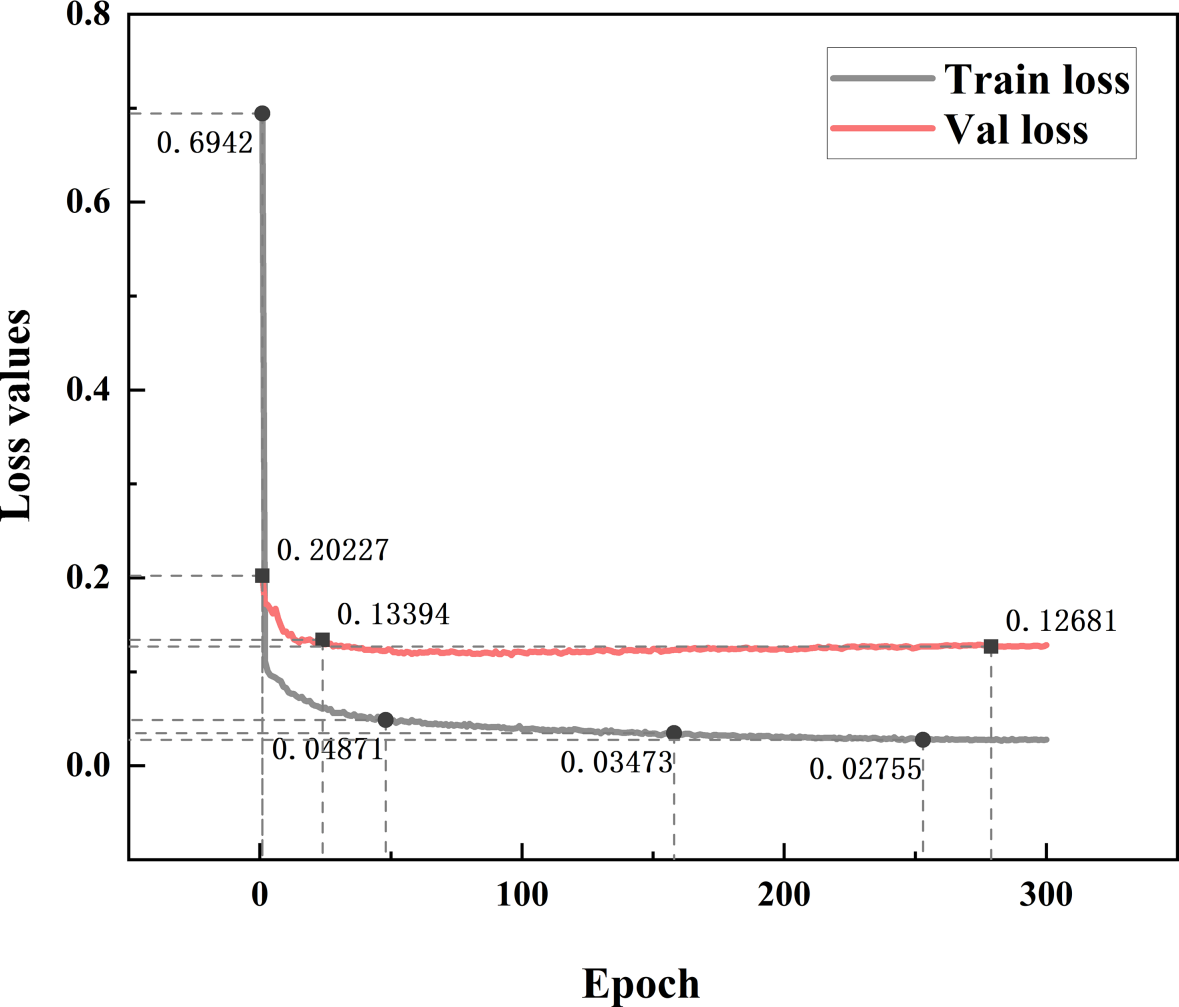
Convergence curve for training dataset and validation dataset.

As shown in **Figure10**, the proposed model TBC-YOLOv7 has been converged after only 10 iterations for the training dataset and the validation dataset, and it exhibits considerable stability. The training loss value varies between 0.02 and 0.1, and the validation loss value varies between 0.1 and 0.16.

Furthermore, the performance comparison between the TBC-YOLOv7 and original YOLOv7 (as shown in **Table 3**) also conclude that TBC-YOLOv7 model clearly superior to YOLOv7 model in terms of tea bud grading recognition. The TBC-YOLOv7 has improved the BOL detection accuracy to 88.2% with an increasement of 6.1%, and the BTL detection accuracy has improved to 86.9%. Meanwhile, both recall rates have also improved. Hence the TBC-YOLOv7 model achieved an AP value of 85% for detecting BTL grades and of 90% for detecting the BOL grades.

**Table 3:** Comparison of experimental results of TBC-YOLOv7 and original YOLOv7.

	Precision (%)		Recall (%)		AP (%)	
Grade	BOL	BTL	BOL	BTL	BOL	BTL
YOLOv7	0.821	0.82	0.804	0.747	0.866	0.817
TBC-YOLOv7	0.882	0.869	0.81	0.759	0.9	0.85

To verify the viability of our proposed improvement method TBC-YOLOv7 in terms of model performance, reflecting the impact of different improvement strategies in the algorithm on the model detection performance, we conducted ablation experiments based on YOLOv7 by incorporating different modules to validate model performance, where '√' indicates that the corresponding model optimization strategy has been added and '-' indicates that it has not been added. In addition, all other training parameters were configured consistently. The results of the ablation experiments are shown in **Table 4**.

**Table 4:** Results of ablation experiments.

Model	CoT	BiFPN	SIOU	CA	Precision (%)	Recall (%)	mAP (%)	F1	FPS
YOLOv7	–	–	–	–	82%	77.6%	84.1%	0.80	8.21
Strategy 1	√	–	–	–	79.9%	79.9%	86.2%	0.80	8.58
Strategy 2	√	√	–	–	83.2%	81.7%	86.7%	0.82	8.32
Strategy 3	√	√	√	–	88%	75.3%	86.9%	0.81	7.96
Strategy 4	√	√	√	√	87.5%	78.4%	87.5%	0.83	7.46

As shown in **Table 4**, strategy 1 is to incorporate the CoT module into the feature extraction network, the model increases 2.1% mAP value over the original model, facilitating feature interactions at different spatial locations and effectively enhancing the recognition of the bud region. Strategy 2 is to incorporate the BiFPN structure of TBC-YOLOv7, which further enhances model accuracy and recall by 1.2% and 4.1%, respectively. This strategy fully fusing different grades of bud features and improving the model’s performance in detecting buds at different scales. Strategy 3 is to integrate the loss function of SIOU into the bounding box regression, the detection accuracy and mAP are up to 88% and 86.9% respectively. It's being attributed to the fact that SIOU can gradually converge prediction box based on the boundary of ground truth box to achieve the effect of overall shape convergence, which can more effectively solve the problem of inconsistent movement direction of the prediction box when the prediction box and target box do not overlap, thereby improving the positioning accuracy of the model bounding box. Strategy 4 is based on strategy 3 by using the CA attention mechanism before feature fusion, the value of mAP has been increased by 3.4%. After feature extraction, the model could focus more on the location information for the intensive detection task, enhancing the saliency of the valuable features of the buds for different scales. From **Table 4**, it can be seen that from strategy 1 to strategy 4, along with the addition of components of CoT, BiFPN, SIOU, CA, the performance indicators have been improved to varying degrees. The accuracy of the TBC-YOLOv7 model has increased by 5.5% compared to the original model, the recall has also increased compared to the original model, and the mAP has improved from 84.1% to 87.5%. Overall, the F1 values of the combined evaluation metrics suggest that the model's overall performance has been optimized to 0.83. The model also has a detection speed of 7.46 FPS. It can be seen that the proposed method in this paper has a particular optimization effect on the detection ability of the YOLOv7 model and can satisfy the practical application requirements.

**Figure 11:**
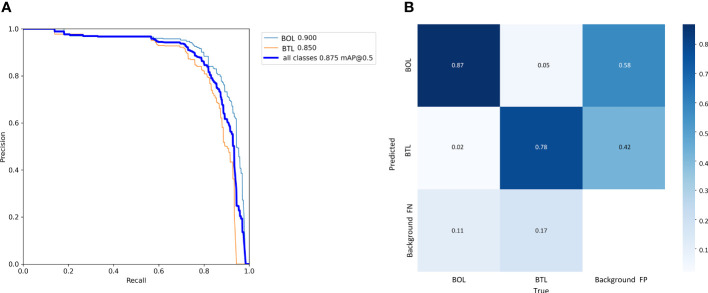
Precision–Recall curve **(A)**: The horizontal axis represents recall, and the vertical axis represents precision. Confusion matrix **(B)**: "BOL" represents the BOL grade, "BTL" represents the BTL grade, and "background" represents the background class. The rows represent the true labels, and the columns represent the predicted classes.

We calculated the precision and recall at different thresholds based on the experimental results, and connected the points to form a PR curve which is shown in **Figure 11A**. The closer the curve is to the top-right corner, the less noticeable the decrease in precision as recall increases, indicating better overall performance of the model. **Figure 11B** presents the confusion matrix summarizing the prediction results for the classification. It can be observed that the TP for the BOL and BTL are 87% and 78%, respectively. The proportion of FP is very small, being 2% and 5%, respectively. The cases of FN in the BTL are more than that of BOL, but the difference between the two is insignificant. The potential reasons for occasional instances of FN may be attributed to a high proportion of occlusions and the influence of complex environmental factors, which can impact the performance of the model. Overall, the classification of tea leaf grades is accurate and comprehensive.

**Figure 12:**
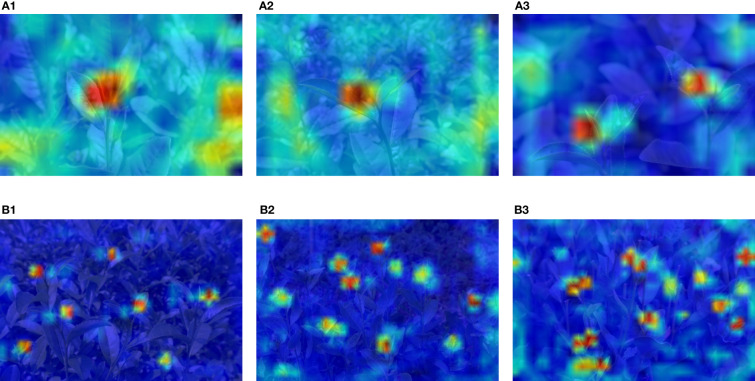
Heat map visualization: **(A1–A3)** with fewer bud targets; **(B1–B3)** with more bud targets. The different color areas of the image represent the level of contribution to the detection.

In order to visualize the region of interest in the tea bud images, a heat map is generated using the Grad-CAM method (as shown in **Figure 12**). The sub-figures of (B1), (B2), and (B3) from **Figure 12** show that the proposed model is capable of precisely identifying small-scale and masked targets, with more precise localization accuracy. It can be seen that the improved mechanism can effectively suppress background noise, further demonstrating that the model has a more robust attention learning capability.

### 4.5 Predicted performance

**Figure 13:**
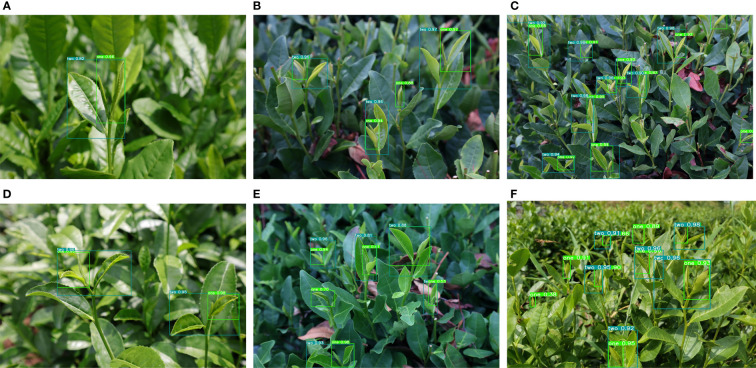
TBC-YOLOv7 detection results: **(A)** for single targets; **(B)** for multi-targets; **(C)** for lower light levels; **(D)** for brighter light levels; **(E)** for intensive situation; **(F)** for targets vary in size.

The prediction results of the TBC-YOLOv7 model for tea bud grading detection are shown in **Figure 13**. For the case of less target detection in **Figure 13A**, the specific locations of tea grades can be accurately labeled with high-confidence results. It can be seen that in the case of strong light intensity in **Figures 13D, F**, even if the surface of the leaves is illuminated by bright light with reflective areas, resulting in a highly similar color of the buds to that of the aged leaves, the TBC-YOLOv7 model can still identify the bud targets and perform the bud grading. What is more, in the case of a large number of bud targets and dense occlusion in **Figures 13C, E**, while the tea buds in the images are affected by the shooting angles and weaken the significance of the features, the model can still accurately identify and grade the smaller bud targets at the edges of the image. These results demonstrate that the optimized TBC-YOLOv7 model significantly improves performance and anti-interference ability in recognizing small targets among multiple targets.

**Figure 14:**
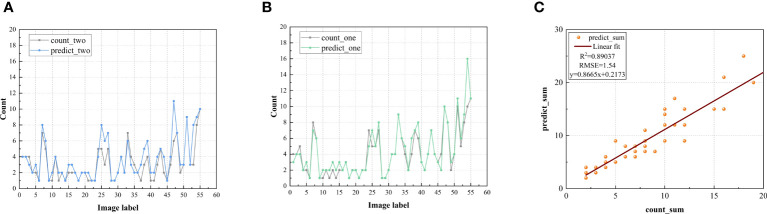
Comparison and evaluation of tea bud grade detection counts: **(A)** predicted number and actual number for BTL; **(B)** predicted number and actual number for BOL; **(C)** linear regression diagram of model between prediction value and actual value.

Based on the proposed model, we compared predicted number and actual number for the two tea bud grades of BOL and BTL (as shown in **Figure 14**). From the sub-figure14 (A) and (B), both the blue and green curves closely approximate the gray curve, where the grey curve represents the actual number of tea buds, the blue curve represents the predicted number of BTL, and the green curve represents the predicted number of BOL. The overall predicted number of tea buds exceeds the actual number of markings, indicating that the model can detect and identify some tea buds that are overly obscured and not marked. However, the difference between the two is insignificant, and the predicted number is very close to the actual number of manual markings. Moreover, we perform a linear regression analysis to assess the correlation between the actual number of manually labeled tea buds and the predicted number of tea buds by the model (as shown in **Figure 14C**, in which the value of $R^{2}\;$ is up to 0.89037, and the value of RMSE is 1.54. These findings indicate that the model's predictions of the number of tea buds are highly correlated with those of the number of artificial tea buds.

### 4.6 Comparison with classical detection algorithms

**Figure 15:**
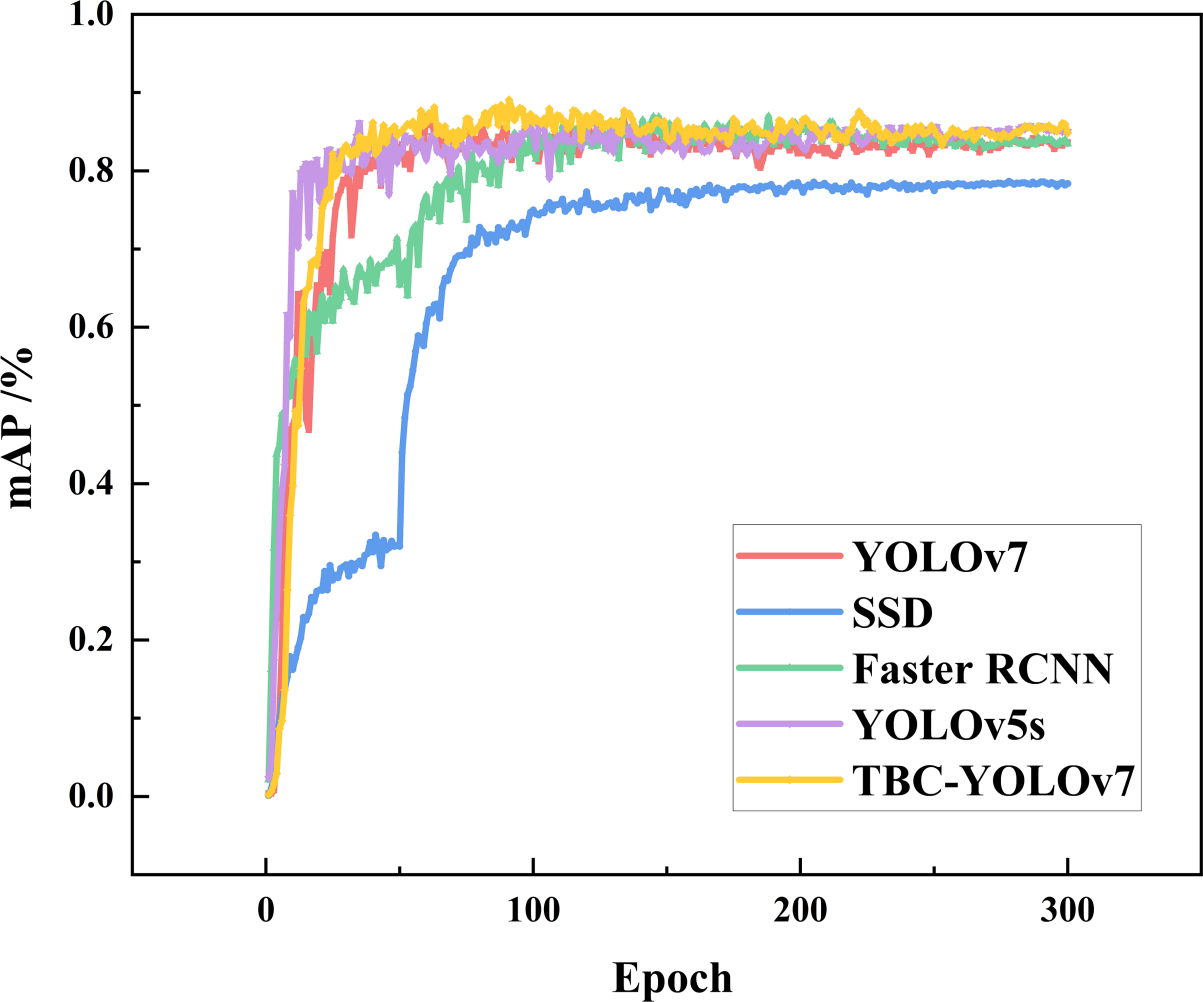
Variation curves of mAP for the models of TBC-YOLOv7, SSD, Faster RCNN, YOLOv5s, and YOLOv7 during training.

To further verify whether the proposed model is superior to classical algorithms in the tea bud detection scenario, TBC-YOLOv7 compared with the mainstream target detection models SSD, Faster RCNN, YOLOv5s, and YOLOv7, and the comparison experiments were all based on the same dataset (as shown in **Figure 15**). The TBC-YOLOv7 model converges faster than the YOLOv7 model as the number of training epochs increases. The mAP value of the TBC-YOLOv7 model after the 80th epoch is higher overall than that of the original YOLOv7, YOLOv5s, SSD, and Faster RCNN.

As shown in **Table 5**, we assess and compare the performance of precision, recall, F1 score, mAP, FPS, FLOPs and parameters. The two-stage detection algorithm, Faster RCNN, achieved an F1 score of 0.7, a mAP of 83.38%, and a 137 MB of parameters. On the other hand, the one-stage detection algorithm, SSD, exhibited improved detection speed compared to Faster RCNN, but its performance suffered when detecting small targets, with mAP and F1 score being 20.65% and 10% respectively, which is significantly lower than TBC-YOLOv7. Comparatively, TBC-YOLOv7 demonstrated a 1.9% and 3.4% increasement of mAP compared to YOLOv5s and YOLOv7, respectively. While ensuring higher accuracy, the FPS of the TBC-YOLOv7 model does not decrease dramatically, and the number of parameters and computation requirements are slightly better than the YOLOv7 model. Considering the superiority of comprehensive performance, compared to other algorithms of SSD, Faster RCNN, YOLOv5s, and YOLOv7, the TBC-YOLOv7 model is more suitable for achieving graded detection of tea buds in realistic scenarios.

**Table 5:** Comparison of performance indicators for the models of TBC-YOLOv7, SSD, Faster RCNN, YOLOv5s, and YOLOv7.

Model	Precision (%)	Recall (%)	mAP	F1	FPS	FLOPs (G)	Parameters (M)
SSD	69.75%	58.66%	66.85%	0.63	41.72	62.75	26.285
Faster R-CNN	60.44%	84.83%	83.38%	0.70	10.53	370.21	137
YOLOv5s	82.8%	79.6%	85.8%	0.81	17.15	15.8	6.69
YOLOv7	82%	77.6%	84.1%	0.80	8.21	103.2	36.4
TBC-YOLOv7	87.5%	78.4%	87.5%	0.83	7.46	100.9	35

**Figure 16:**
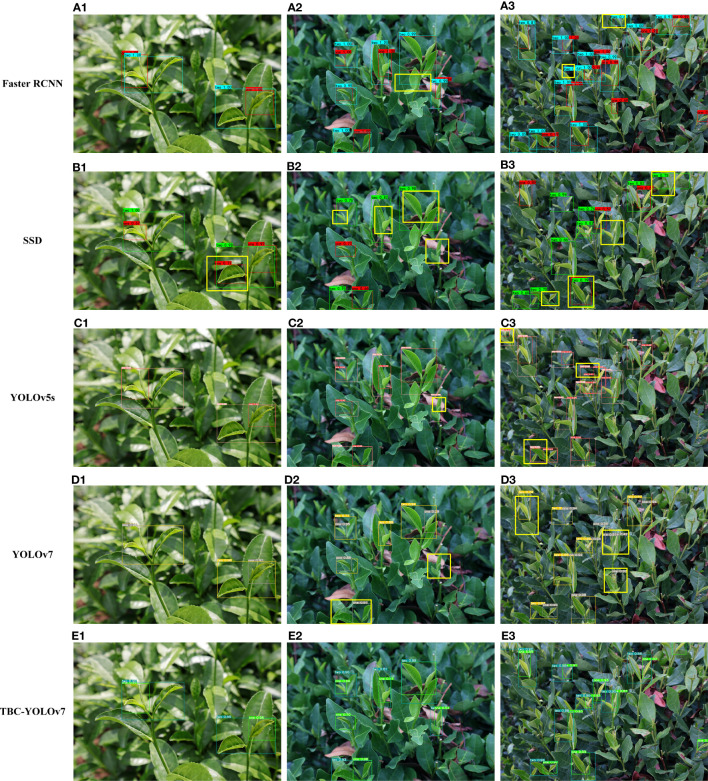
Visualization results predicted by the five models: **(A)** Faster RCNN; **(B)** SSD; **(C)** YOLOv5s; **(D)** YOLOv7; **(E)** TBC-YOLOv7. **(A1–E1)** shows the detection of multiple tea buds under low light conditions. **(A2–E2)** illustrate the detection of tea buds with multiple targets under weak light conditions. **(A3–E3)** show the dense target recognition under uniform illumination conditions. Differences in detection results for each algorithm are marked with bold yellow boxes.

In this study, we evaluated the detection performance of five models under natural practical conditions (as shown in **Figure 16**). As observed, due to the obvious characteristics of the buds and the small number of targets in **Figure 16 A1-E1**, all algorithms can detect correctly except the SSD algorithm with one false detection. In **Figure 16 A2-E2**, the prediction boxes of the Faster RCNN algorithm appear out of position, the SSD algorithm has multiple missed detections, the YOLOv5s algorithm has one missed detection in the image, and the YOLOv7 algorithm can accurately detect the buds but has apparent errors in the discrimination of the grade, and the TBC-YOLOv7 model can accurately identify the tea grade even in the case of blurred images. **Figure 16 A3-E3** shows the dense target recognition under uniform illumination. The SSD algorithm has a large number of false detections, the YOLOv5s algorithm has false detections at the edges of the image and in the blurred areas, and the YOLOv7 algorithm incorrectly classifies the densely shaded bud target in the center of the image, resulting in two prediction boxes on a BOL target, while the Faster RCNN algorithm identifies the tea buds that only appear halfway at the edges of the image as BTL. In contrast, The TBC-YOLOv7 model solved the problem of false detection caused by inconspicuous features and achieved a certain improvement in accuracy compared with other models.

## 5 Discussion

Traditional manual tea picking methods currently produce the disadvantages, including low picking efficiency, and excessive reliance on manual working experience judgment. The density growth if tender leaves, as well as the color similarity among buds, tender leaves, and old leaves, are key factors affecting rapid and high-precision grading. Therefore, there are challenges in accurately identifying tea buds. In practical scenarios, due to various factors such as dense tea leaves, a small proportion of tea buds, weak feature saliency, and partial occlusion, the feature information of small target objects is easily lost after a large amount of convolution during the feature extraction process of the detection network. In order to overcome the difficulties of this detection, the present study introduces a model capable of accurately identifying tea buds and achieving more precise grading. The TBC-YOLOv7 model outperforms the other models under various challenging conditions, such as multiple targets, complex backgrounds, and multiple scales. Furthermore, compared with mainstream algorithms, the TBC-YOLOv7 reduces the false detection rate caused by unclear features of different tea bud grades and achieve a certain degree of improvement in accuracy, laying a theoretical foundation for intelligent tea picking machinery. By detecting the grade of tea buds, it ensures that tea producers can precisely control the quality of tea and ensure that different grades of tea are valued correctly in the market. Currently, there is limited research on tea bud grading detection. Wang et al. (2023) only considered using the YOLOv7 detection algorithm for tender bud detection, with a small image field of view. Chen & Chen (2020) utilized the Faster RCNN algorithm to extract tea buds and picking points, but their recognition performance is not as good as the method proposed in this paper.

In practical automated harvesting, it is not only necessary to focus on the recognition accuracy of the picking robot but also consider the success rate of harvesting. Additionally, in the real-world environment, challenges such as pixel blurring may occur during the movement process, leading to the shaking and compression of tea bud targets. Moreover, there can be interference caused by changes in tea bud poses, making the scenario more complex than the original scenario. To address these issues, future efforts should incorporate sensors for accurate localization of picking points. This can help in handling tea bud detection under severe shadow conditions and reducing interference during motion recognition (Hong et al., 2023). There is a scarcity of research that establishes the general adaptability of different types of tea leaves in this research. It is crucial to obtain a wider range of image data to consider subtle differences between different varieties. This will enhance the universality of the detection model.

## 6 Conclusion

With the development of agricultural informatization, the identification of tea raw materials is changing from traditional manual evaluation methods to automated intelligent grading methods. This paper proposed the TBC-YOLOv7 model. In this model, the CoT can provide higher quality feature extraction capabilities. The BiFPN and the SIOU loss were used to enhance the transfer of feature information between different network layers to achieve a deeper level of integration by integrating the local and global features of the tea buds. The CA further utilizes more shallow features to more accurately locate and recognize interested targets.

Overall, the proposed model improves the results on tea bud detection and grading in natural environment, meanwhile it can improve the positioning accuracy. The TBC-YOLOv7 achieves a mAP value of 87.5% with an increasement accuracy of 5.5%. Moreover, the F1 score is up to 0.83. The regression analysis of the model also reflects a high degree of correlation ( $R^{2}\;$ = 0.89) and a low RMSE of 1.54. The study confirms that integrating the transformer-based self-attentive module into the target detection model can improve the detection accuracy of tea bud targets and outperform other classical models. Consequently, the proposed model provides a technical support for the accurate detection of tea buds in the actual natural environment. Subsequent research will focus on deploying the model for real-time detection on mobile devices. Moreover, integrating application interfaces with existing tea production processes and systems, as well as providing remote access and management capabilities for large-scale production scenarios. In the future, the application scenarios can be expanded to provide references for other detection solutions, such as flower detection and fruit detection, thereby offering new research pathways for core technologies in smart agriculture.

## Data availability statement

The raw data supporting the conclusions of this article will be made available by the authors, without undue reservation.

## Author contributions

Conceptualization, DW. Formal analysis, DW. Funding acquisition, DW. Methodology, XZ. Resources, SW. Writing—original draft, SW All authors have read and agreed to the published version of the manuscript. All authors contributed to the article and approved the submitted version.
